# A neonate with giant omphalocele complicated by mechanical intestinal obstruction requiring left hepatic lobe resection: a case report

**DOI:** 10.3389/fped.2026.1895051

**Published:** 2026-07-23

**Authors:** Jingjing Gong, Jing Li, Zongfang Teng, Juan Pei, Aixiang Li

**Affiliations:** Linyi People’s Hospital, Linyi, China

**Keywords:** case report, giant omphalocele, left hepatic lobe resection, mechanical intestinal obstruction, neonate, staged surgical management

## Abstract

**Background:**

Giant omphalocele is a rare congenital abdominal wall defect often associated with viscero-abdominal disproportion and frequently requiring prolonged staged repair. Secondary mechanical bowel obstruction during staged reduction is uncommon and may be difficult to distinguish from expected neonatal dysmotility or postoperative ileus. Reports of hepatic resection to facilitate reduction in neonates with giant omphalocele are extremely limited.

**Case presentation:**

We report a term small-for-gestational-age female neonate with a giant liver-containing omphalocele. Initial management included intensive care support, sac protection, ventilatory support, and staged reduction by sac suspension. On hospital day 15, new bilious or turbid gastric drainage and deterioration of the omphalocele sac prompted urgent reassessment for secondary bowel compromise. Because the infant was clinically unstable, urgent operative exploration was prioritised over additional radiological investigations. Exploratory laparotomy revealed yellow fluid within the sac, dense adhesions involving the small bowel, greater omentum, and liver, and adhesion bands causing small-bowel kinking. Congenital intestinal malrotation was also present, but no frank bowel necrosis was identified. Complete reduction remained unfeasible, and a sterile silo pouch was placed for continued staged management. Despite continued staged treatment, meaningful further reduction could not be achieved because of extremely limited abdominal domain, marked visceral oedema, and the large non-compressible herniated liver. On hospital day 21, second-stage reduction with left hepatic lobe resection was performed as an exceptional rescue strategy after conventional staged reduction had failed. At 6 months’ follow-up, the patient was tolerating oral feeding and showed age-appropriate growth and neurodevelopment, with no clinically evident ventral hernia or liver-related complications.

**Conclusion:**

Neonates with giant omphalocele who undergo staged treatment and develop new bilious or turbid gastric drainage together with deterioration of the omphalocele sac require urgent assessment for mechanical bowel obstruction. Timely reassessment, flexible staged surgery, and multidisciplinary intensive care are critical to successful management. In highly selected cases with persistent severe loss of abdominal domain after failure of conventional staged reduction, hepatic resection may be considered, if at all, only as an exceptional last-resort rescue strategy.

## 1 Introduction

Congenital omphalocele is a rare but severe abdominal wall defect characterized by failure of the midgut to return into the abdominal cavity, resulting in herniation of abdominal organs covered solely by a thin, transparent, and fragile membrane. The incidence is approximately 1/6,000–1/4,000 live births (1). Clinically, omphaloceles are classified into minor, giant, and ruptured types, depending on the size of the herniation and the integrity of the covering membrane. Giant omphalocele is commonly defined as an abdominal wall defect larger than 5 cm and/or herniation containing a substantial portion of the liver (2, 3). Omphalocele is frequently associated with chromosomal abnormalities, syndromic conditions, and other structural anomalies, and prenatal and postnatal factors may influence neonatal outcomes (4, 5). These infants often present major management challenges because of loss of abdominal domain, respiratory compromise, risk of sac rupture, fluid and heat loss, infection and delayed establishment of enteral feeding (6).

Management of giant omphalocele is highly individualised and often requires staged reduction rather than primary closure, especially when immediate reduction may increase intra-abdominal pressure and compromise cardiorespiratory function (2, 3, 7). However, the clinical course may be complicated by gastrointestinal deterioration, and distinguishing evolving mechanical intestinal obstruction from expected neonatal dysmotility or postoperative ileus can be difficult in critically ill neonates (6, 8). Here, we report a neonate with a giant liver-containing omphalocele who developed mechanical intestinal obstruction during staged management. This case highlights the importance of distinguishing secondary bowel obstruction from expected dysmotility and illustrates the need for flexible, multidisciplinary, staged surgical planning. In addition, this case is unusual because persistent severe loss of abdominal domain ultimately necessitated resection of the left hepatic lobe as a non-routine rescue measure to facilitate further reduction. Contemporary literature on giant omphalocele provides very limited discussion of hepatic resection as a strategy to facilitate visceral reduction in neonates.

## 2 Case presentation

### 2.1 Patient information and clinical findings

A female neonate delivered by caesarean section at 39 weeks’ gestation was admitted to our neonatal intensive care unit shortly after birth. Prenatal imaging and detailed maternal obstetric records were unavailable at the time of retrospective case preparation. Her Apgar scores were 9 at 1 min and 10 at 5 min. Birth weight was 2090g, birth length was 44 cm, and head circumference was 31 cm, consistent with small for gestational age status.

On admission, her temperature was 36.0 °C, pulse rate was 128 beats/min and respiratory rate was 45 breaths/min. Physical examination revealed a giant omphalocele measuring approximately 15 × 20 cm, with an intact sac containing the liver and intestinal loops and covering almost the entire abdominal region. She was admitted to the neonatal intensive care unit for urgent multidisciplinary management.

### 2.2 Timeline

The major clinical events and interventions are summarised in Table 1.

**Table 1:** Timeline of the clinical course and management.

Hospital day	Clinical event	Management
Hospital day 1	Admission with giant omphalocele	NICU admission, mechanical ventilation, nil by mouth, gastrointestinal decompression, TPN, antibiotics, sac protection, multidisciplinary team (MDT) assessment
Hospital day 1	Initial operative management	Omphalocele sac suspension
Hospital day 15	Yellow-green turbid drainage and worsening sac condition, concern for mechanical intestinal obstruction	Emergency MDT review and exploratory laparotomy with adhesiolysis, sac membrane excision and artificial silo placement
Hospital day 21	Second-stage surgical reduction after multidisciplinary reassessment	Second-stage reduction with exploratory laparotomy, adhesiolysis, left hepatic lobe resection, and omphalocele reduction
Postoperative period	Gradual recovery	Weaning from invasive to non-invasive ventilation, careful wound care, nutritional support, monitoring of bowel function
Late hospital stay	Gastrointestinal recovery	Discontinuation of decompression, initiation and advancement of enteral feeding, transition to oral feeding
Hospital day 90	Clinical stabilisation	Transfer to general ward and discharge shortly afterwards
6-month follow-up	Follow-up	Oral feeding tolerated; satisfactory growth and age-appropriate neurodevelopment; no clinically evident ventral hernia or liver-related complication

### 2.3 Diagnostic assessment

At admission, the diagnosis of giant omphalocele was established clinically on the basis of physical examination. The sac was intact and contained both liver and bowel. Urinary tract ultrasonography showed normally positioned kidneys, with only mild separation of the right renal pelvis, approximately 4 mm, without ureteric dilatation; an urachal cystic echo was also observed. Echocardiography showed mild tricuspid regurgitation, mild aortic regurgitation, moderate pulmonary hypertension, patent ductus arteriosus and patent foramen ovale. Repeat echocardiography confirmed the presence of patent ductus arteriosus and patent foramen ovale. During hospitalisation, bedside monitoring focused on respiratory status, abdominal tension, bowel activity, wound and sac condition, and signs of infection. On hospital day 15, worsening yellow-green turbid gastrointestinal decompression output and deterioration of the sac surface raised concern for secondary mechanical intestinal obstruction. The differential diagnosis included postoperative ileus or gastrointestinal dysmotility, bowel ischaemia, sepsis-related intestinal dysfunction, and malrotation with volvulus. However, progressive bilious or turbid gastric drainage together with deterioration of the omphalocele sac was considered atypical for simple dysmotility alone.

Because the infant was clinically unstable, additional abdominal radiography or ultrasonography was deferred, and urgent operative exploration was prioritised to avoid treatment delay. Operative findings of dense adhesions involving the small bowel, greater omentum, and liver, together with adhesion bands causing small-bowel kinking and congenital intestinal malrotation, supported the diagnosis of mechanical intestinal obstruction without frank bowel necrosis. Formal preoperative volumetric assessment of the liver-to-abdominal cavity relationship was not available; operative decision-making relied on serial clinical reassessment and intraoperative findings.

### 2.4 Therapeutic intervention

On hospital day 1, multidisciplinary consultation was performed to determine the management strategy. Omphalocele sac suspension was performed by the paediatric surgical team. The procedure involved gentle support and elevation of the sac without excessive pressure. The infant received mechanical ventilation, nil-by-mouth management, gastrointestinal decompression, total parenteral nutrition, and intravenous piperacillin-tazobactam. The sac was covered with sterile plastic wrap and warm saline-soaked dressings to maintain membrane moisture and reduce heat loss, fluid loss, and infection risk (2).

On hospital day 15, yellow-green turbid fluid was noted from gastrointestinal decompression, and the surface of the protruding omphalocele sac appeared abnormal and incomplete. Because of rapid clinical deterioration and concern for secondary bowel compromise, emergency multidisciplinary reassessment was undertaken. As the infant was clinically unstable, additional abdominal radiography or ultrasonography was deferred, and urgent operative exploration was prioritised to avoid treatment delay. Intraoperatively, yellow fluid was observed within the omphalocele sac. Exploratory laparotomy revealed dense adhesions between the small bowel, greater omentum, and liver. Adhesion bands caused kinking of the small bowel, and congenital intestinal malrotation was also present. No bowel necrosis, perforation, internal hernia, or clear volvulus was identified. The adhesion bands contributing to mechanical obstruction were released, the intestinal loops were realigned, and the abdominal cavity was irrigated until clear fluid was obtained. The liver was enlarged, and complete reduction of the small bowel and liver remained difficult because of limited abdominal domain. Therefore, the omphalocele sac membrane was excised, and a sterile silo pouch was sutured to the skin. The remaining intestinal loops and liver were placed into the sterile silo pouch, which was then covered with ointment and gauze. The infant was subsequently transferred back to the neonatal intensive care unit for continued postoperative care.

Despite continued staged reduction with silo-based management, no meaningful further reduction could be achieved over the following days. The persistent failure of reduction was attributed to severe viscero-abdominal disproportion, marked visceral oedema, and the large herniated liver, which together prevented safe return of the abdominal contents. After repeated multidisciplinary reassessment, prolonged continuation of silo treatment alone was judged unlikely to achieve timely closure or safe reduction.

On hospital day 21, the patient underwent exploratory laparotomy with adhesiolysis, left hepatic lobe resection, and staged reduction of the giant omphalocele. Intraoperative findings showed an existing artificial silo pouch covering the prolapsed visceral organs. A 9-cm abdominal wall defect extended from the xiphoid process superiorly to 3 cm above the pubic bone inferiorly. After removal of the artificial silo pouch, the small intestine, liver, spleen, pancreas, and stomach were found to be herniated through the abdominal wall defect. The herniated visceral organs were oedematous and adherent. Congenital intestinal malrotation was again noted, and the liver appeared as a spherical mass measuring approximately 8 × 6 × 6 cm. After evacuation of gastrointestinal contents, the intestinal volume did not decrease sufficiently to allow safe reduction. The left hepatic lobe was judged intraoperatively to be a major non-compressible barrier to further reduction. Because the abdominal cavity remained critically limited despite prior staged management and intraoperative decompressive manoeuvres, left hepatic lobe resection was undertaken as an exceptional rescue measure rather than a routine component of repair. The resection plane was identified between the left and right hepatic lobes, and left hepatic lobe resection was performed. Haemostasis was achieved by vascular ligation and electrocautery. The tissues surrounding the abdominal wall defect were dissected to increase abdominal capacity and facilitate reduction. Next, the right hepatic lobe, stomach, spleen, and colon were repositioned in the abdominal cavity. Because complete reduction of the small bowel remained unsafe due to limited abdominal capacity, the small bowel was placed in a silo pouch for continued staged reduction. After haemostasis was confirmed and no leakage from the silo pouch was observed, a sterile dressing was applied. There were no complications during the surgery, and the estimated blood loss was 3 mL. The infant was transferred to the neonatal intensive care unit with an endotracheal tube in place and stable vital signs. Postoperatively, she was nursed in an incubator with the silo pouch suspended and underwent close clinical monitoring.

Postoperatively, recovery was favourable. Ventilation was gradually transitioned from invasive to non-invasive support. Nasogastric decompression was discontinued, and minimal enteral feeding was introduced with careful monitoring of tolerance. Feeding volumes were progressively increased, and the infant successfully transitioned to oral feeding with spontaneous bowel movements and adequate urine output.

**Figure 1:**
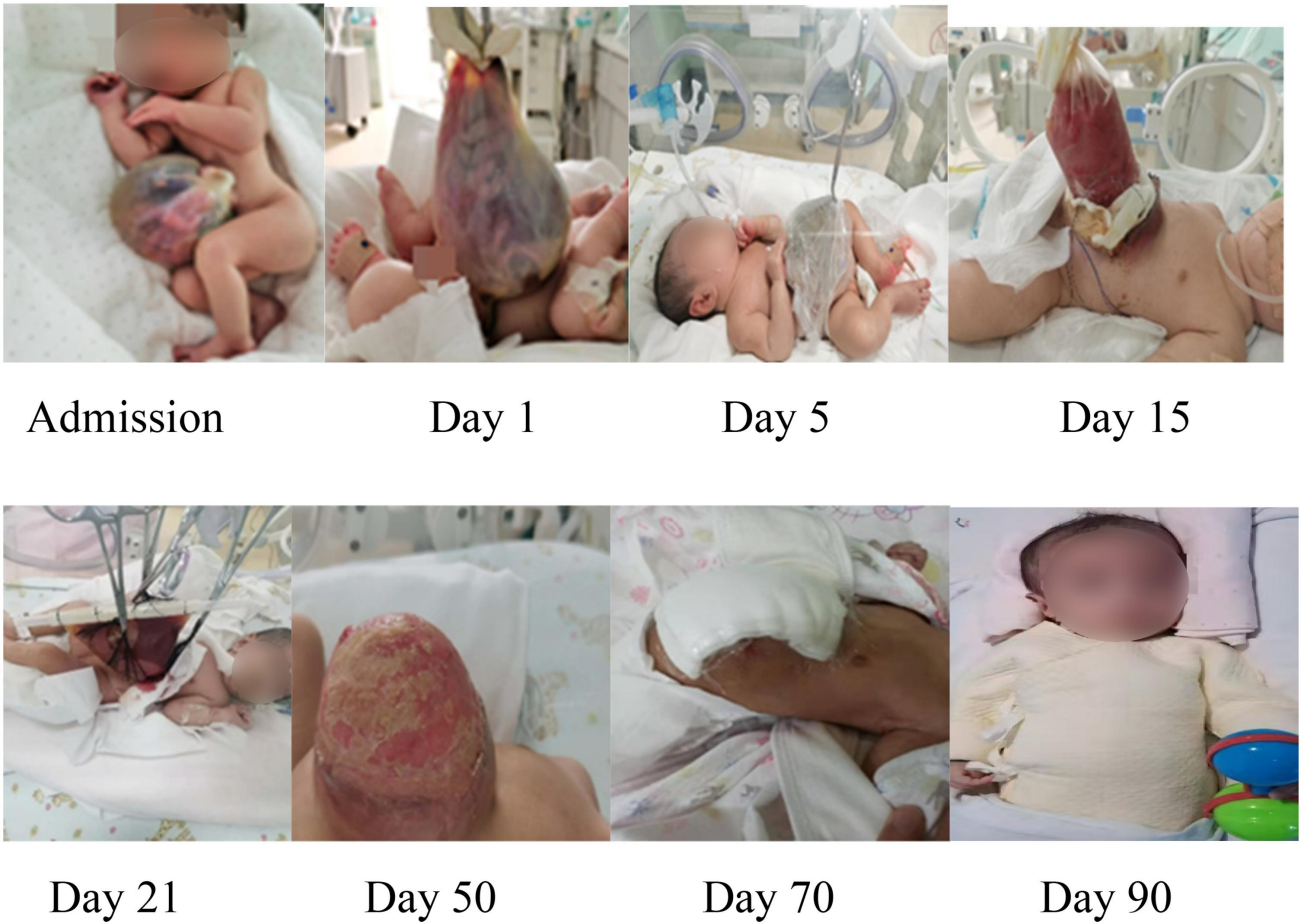
Clinical progression of the giant omphalocele during hospitalisation, showing the appearance at admission and on hospital days 1, 5, 15, 21, 50, 70, and 90. Potentially identifying features were obscured using uniform masking to protect patient privacy.

On hospital day 90, the infant was transferred from the neonatal intensive care unit to the general ward in stable condition and was discharged shortly thereafter. The clinical progression of the giant omphalocele during hospitalisation is shown in Figure 1.

### 2.5 Follow-up and outcomes

At 6-month follow-up, the infant was tolerating oral feeding, showed satisfactory interval growth and age-appropriate neurodevelopment on clinical assessment, and had no clinically evident ventral hernia, recurrent bowel obstruction, jaundice, abdominal distension, or other liver-related complication during this short-term follow-up period.

## 3 Discussion

### 3.1 Recognition of secondary mechanical intestinal obstruction

Giant omphalocele remains a challenging congenital abdominal wall defect without a universally accepted management pathway (2). It presents multiple simultaneous challenges, including limited abdominal capacity, risk of sac injury, respiratory compromise, infection, delayed feeding, and prolonged dependence on intensive care (8). This case illustrates the complexity of managing giant omphalocele in a critically ill neonate, particularly when the clinical course is complicated by mechanical intestinal obstruction. In neonates with giant omphalocele, delayed gastrointestinal transit and feeding intolerance may be expected during prolonged intensive care (8); however, in our patient, the development of new yellow-green turbid gastric drainage together with visible deterioration of the sac was considered atypical and prompted urgent reassessment. The intraoperative findings of adhesions, adhesive bands, and intestinal malrotation demonstrated that the clinical deterioration was attributable to mechanical obstruction rather than expected dysmotility alone. This finding highlights the importance of serial clinical examination throughout staged management.

### 3.2 Multidisciplinary and staged surgical management

Multidisciplinary care was essential in this case and has been recognised as a key component of giant omphalocele management, particularly for operative timing, respiratory assessment, nutritional support, wound care, and postoperative monitoring (2). Throughout the hospitalisation, coordinated care by neonatologists, paediatric surgeons, anaesthesiologists, and specialist nurses enabled early detection of clinical deterioration, timely reassessment, perioperative optimisation, respiratory support, nutritional management, and postoperative wound care.

Staged surgical management was another key component of treatment. Immediate primary closure of giant omphalocele can be unsafe when abdominal capacity is severely limited, because forced reduction may increase intra-abdominal pressure and compromise cardiorespiratory function (7). In the present case, initial sac suspension followed by silo-based management allowed gradual reduction and ongoing reassessment. However, the development of mechanical bowel obstruction required urgent operative intervention. Even after adhesiolysis and continued silo management, further reduction remained unsuccessful because of severe viscero-abdominal disproportion, marked visceral oedema, and the large non-compressible herniated liver.

### 3.3 Left hepatic lobe resection as an exceptional rescue strategy

The most distinctive aspect of this case was the exceptional use of left hepatic lobe resection to facilitate reduction after failure of conventional staged management. This intervention was not planned as a standard component of repair, but was considered only after conventional staged reduction and intraoperative decompressive manoeuvres failed to achieve safe reduction. Formal volumetric imaging was not available because the infant was clinically unstable and urgent operative decision-making was required. The decision therefore relied on serial bedside reassessment and intraoperative judgement, which indicated that the large herniated left hepatic lobe constituted a major non-compressible barrier to reduction. In this context, left hepatic lobe resection was undertaken as an exceptional rescue measure to gain additional abdominal domain. Continuation of silo-based management alone was considered but was judged unlikely to achieve safe or timely closure because reduction had plateaued despite ongoing treatment, visceral oedema remained marked, and the herniated liver continued to function as a fixed non-compressible barrier. Further delay also risked prolonging ventilatory support, parenteral nutrition, and exposure to additional bowel compromise or infection. On this basis, hepatic resection was selected as an individualised rescue measure rather than as an extension of routine staged management.

Only isolated reports have described hepatic resection or liver-size reduction in the context of omphalocele repair. Kleinhaus et al. reported partial hepatectomy during omphalocele repair in 1968 (9). Delarue et al. later described delayed reconstruction of an omphalocele-related ventral hernia requiring abdominosternoplasty, right hepatectomy, and partial splenectomy in an adolescent patient (10). These reports differ substantially from the present neonatal case, but they support the view that hepatic resection has been used only rarely and in highly selected circumstances. In the literature reviewed for this case, we did not identify established evidence-based indications for hepatic resection to facilitate reduction in neonates with giant omphalocele. Published literature on giant omphalocele has mainly focused on staged closure techniques, delayed non-operative approaches, and respiratory outcomes (3, 8). Accordingly, the present case should not be interpreted as support for routine hepatic resection in giant omphalocele. Rather, it illustrates that, in highly selected patients with persistent severe loss of abdominal domain after failure of conventional staged strategies, hepatic resection may rarely be considered as an individualised rescue option. Its potential use must be weighed carefully against the risks of hepatic bleeding, biliary complications, and uncertainty regarding longer-term hepatic outcomes. The novelty of this case lies not in staged management itself, but in the use of left hepatic lobe resection as an individualised rescue strategy after failure of conventional staged reduction in the setting of persistent severe loss of abdominal domain.

### 3.4 Limitations and concluding remarks

The main limitation of this report is that it describes a single patient; therefore, the findings cannot be generalised to all neonates with giant omphalocele. Chromosomal or genetic testing was not performed, despite the known association of omphalocele with chromosomal abnormalities, syndromic conditions, and other structural malformations (4). Accordingly, an underlying genetic diagnosis cannot be excluded. Another limitation is the absence of formal preoperative volumetric imaging to quantify the relationship between liver volume and abdominal domain. Because the infant was clinically unstable, urgent operative management was prioritised over additional imaging, and decision-making relied on dynamic clinical reassessment and intraoperative findings. We also did not identify established published criteria supporting hepatic resection to facilitate reduction in giant omphalocele; therefore, the rationale for this intervention in the present case was necessarily based on dynamic clinical assessment and intraoperative judgement rather than validated selection standards. Finally, follow-up was limited to 6 months and did not include objective post-discharge imaging, laboratory assessment of hepatic function, or standardised neurodevelopmental testing; therefore, longer-term abdominal wall, nutritional, neurodevelopmental, and hepatic outcomes remain to be established. Nevertheless, this case suggests that a favourable short-term outcome may be achievable in a neonate with giant omphalocele complicated by mechanical intestinal obstruction when staged surgery is combined with coordinated multidisciplinary perioperative care.

Despite these limitations, this case emphasises that new bilious or turbid gastric drainage with deterioration of the omphalocele sac during staged management should prompt urgent reassessment for secondary mechanical intestinal obstruction. It also suggests that hepatic resection, if considered at all, should be reserved for highly selected rescue situations after failure of conventional staged reduction, rather than being regarded as a routine component of giant omphalocele repair.

## 4 Conclusion

This case highlights that mechanical intestinal obstruction may arise during staged management of giant omphalocele and may initially mimic expected neonatal gastrointestinal dysmotility. New bilious or turbid gastric drainage, together with deterioration of the omphalocele sac, should prompt urgent reassessment for secondary bowel compromise. Successful treatment may require flexible staged operative planning. When conventional staged reduction fails because of persistent severe loss of abdominal domain, hepatic resection should be considered, if at all, only as an exceptional and non-routine rescue strategy in highly selected patients.

## Data Availability

The datasets presented in this article are not readily available because of ethical and privacy restrictions. Requests to access the datasets should be directed to the corresponding author.

## References

[1] Bhatt P Poku FA Umscheid J Ayensu M Parmar N Vasudeva R. Trends in prevalence and mortality of gastroschisis and omphalocele in the United States from 2010 to 2018. World J Pediatr WJP. (2022) 18(7):511–4. 10.1007/s12519-022-00544-2 35294711

[2] Saxena AK Hayward RK Mutanen A Goneidy A Ghattaura H Gorter R. European Paediatric Surgeons’ Association consensus statement on the management of giant omphalocele. Eur J Pediatr Surg. (2025) 35(05):407–16. 10.1055/a-2590-5592 40389219

[3] Ghattaura H Ross A Aldeiri B Mutanen A Saxena A. Managing giant omphalocele: a systematic review of surgical techniques and outcomes. Acta Paediatr. (2024) 113(11):2459–65. 10.1111/apa.17346 38992931

[4] Al Namat D Roșca R Al Namat R Hanganu E Ivan A Hînganu D. Omphalocele and associated anomalies: exploring pulmonary development and genetic correlations-a literature review. Diagnostics. (2025) 15(6):675. 10.3390/diagnostics15060675 40150018 PMC11940968

[5] Namat DA Hînganu D Roșca AR Lozneanu L Țarcă E Namat NA. Prenatal and postnatal determinants of outcome in neonates with omphalocele: a 25-year single-center cohort study. J Clin Med. (2026) 15(10):3615. 10.3390/jcm15103615 42194576 PMC13206850

[6] Baerg JE Munoz AN. Long term complications and outcomes in omphalocele. Semin Pediatr Surg. (2019) 28(2):118–21. 10.1053/j.sempedsurg.2019.04.004 31072460

[7] Skarsgard ED. Immediate versus staged repair of omphaloceles. Semin Pediatr Surg. (2019) 28(2):89–94. 10.1053/j.sempedsurg.2019.04.010 31072464

[8] Martou L Saxena AK. Morbidity in giant omphaloceles: predictive factors and management strategies. Acta Paediatr. (2024) 113(12):2504–12. 10.1111/apa.17387 39115971

[9] Kleinhaus S Kaufer N Boley SJ. Partial hepatectomy in omphalocele repair. Surgery. (1968) 64(2):484–5.PMID: 5673064. 5673064

[10] Delarue A Camboulives J Bollini G Bardot J Guys JM. Delayed cure of an omphalocele requiring abdominosternoplasty, right hepatectomy and partial splenectomy. Eur J Pediatr Surg. (2000) 10(1):58–61. 10.1055/s-2008-1072325 10770250

